# Respiratory Syncytial Virus Infections in Infants Affected by Primary Immunodeficiency

**DOI:** 10.1155/2014/850831

**Published:** 2014-06-25

**Authors:** Marcello Lanari, Silvia Vandini, Maria Grazia Capretti, Tiziana Lazzarotto, Giacomo Faldella

**Affiliations:** ^1^Pediatrics and Neonatology Unit, Imola Hospital, Via Montericco 4, 40026 Imola, Italy; ^2^DIMEC, Neonatology and Neonatal Intensive Care Unit, St. Orsola-Malpighi Hospital, Via Massarenti 11, University of Bologna, 40138 Bologna, Italy; ^3^DIMES, Clinical Microbiology Unit, Laboratory of Virology, St. Orsola-Malpighi Hospital, Via Massarenti 9, University of Bologna, Bologna, Italy

## Abstract

Primary immunodeficiencies are rare inherited disorders that may lead to frequent and often severe acute respiratory infections. Respiratory syncytial virus (RSV) is one of the most frequent pathogens during early infancy and the infection is more severe in immunocompromised infants than in healthy infants, as a result of impaired T- and B-cell immune response unable to efficaciously neutralize viral replication, with subsequent increased viral shedding and potentially lethal lower respiratory tract infection. Several authors have reported a severe clinical course after RSV infections in infants and children with primary and acquired immunodeficiencies. Environmental prophylaxis is essential in order to reduce the infection during the epidemic season in hospitalized immunocompromised infants. Prophylaxis with palivizumab, a humanized monoclonal antibody against the RSV F protein, is currently recommended in high-risk infants born prematurely, with chronic lung disease or congenital heart disease. Currently however the prophylaxis is not routinely recommended in infants with primary immunodeficiency, although some authors propose the extension of prophylaxis to this high risk population.

## 1. Introduction

RSV is a ubiquitous RNA virus of the* Pneumovirus* genus and Paramyxoviridae family responsible for frequent acute respiratory infections especially in young infants. Specific antibodies are detectable in 87% of infants younger than 18 months [1] and virtually in all 3-year-old infants.

It was estimated that over 33 million episodes of RSV-related lower respiratory tract infections (LRTI) occurred worldwide in 2005 in children younger than 5 years of age [2]. During that same year, the estimated hospitalizations for severe acute LRTI in young children were 3.4 (2.8–4.3) million (16.9 per 1000 for infants aged 0 to 5 months and 5.1 per 1000 for infants aged 6 to 11 months). The mortality rate was 66,000–199,000/year for children younger than 5 years; 99% of deaths occurred in the developing countries [3].

RSV epidemics in infants younger than 5 years old have a strong impact on pediatric healthcare. In 2000 in the USA, RSV infections lead to 86,000 hospitalizations, 402,000 emergency room visits, 1.7 million office visits, and 236,000 hospital outpatient visits. The estimated cost of these visits and hospitalization was near $258 million [4]. Moreover, RSV LRTI was observed to have a long term impact in the incidence of recurrent wheezing during the first year of life, as recently pointed out in healthy preterm infants born at 33–35 weeks of gestational age [5].

Although RSV-associated mortality is ninefold higher than influenza-associated mortality during the first year of life [6], mortality rate in healthy infants with RSV infections is less than 0.5%, but it may reach up to 60% in infants with immunodeficiency [7]. Several studies have reported that life-threatening RSV LRTI are more frequent in infants with congenital or acquired immunodeficiencies (HIV infections, hematopoietic stem cells, and solid organs transplant recipients) than in healthy infants [8–12]. These infants have prolonged viral shedding because of impaired B-cell and T-cell immunity, lymphopenia, and neutropenia [13]. Prolonged viral shedding may lead to a higher viral load, which is responsible for a more severe LRTI.

## 2. Immune Response to RSV Infections in Infancy

Two different subtypes (A and B) of RSV were identified depending on variations in G protein and the two subtypes coexist during every RSV season. Subtype A is probably associated to more severe infections [14, 15]. The severity of the disease and the need for hospitalization are not only related to the subtype, but also to individual factors influencing the immune response such as age, prematurity, preexisting diseases, immunological disorders, and environmental factors (birth during the epidemic season, presence of siblings, crowding, second-hand smoke exposure, daycare attendance, pollutions, meteorological parameters as temperature, and humidity that may interfere with viral replication and with the clearance of the virus in the airways [16–18]).

Similar to other respiratory viruses, RSV infection does not induce an effective immunological memory because of the high heterogeneity of protein A, B, and G [19] and for this reason it can cause reinfections that are usually less severe than the primary infection and may occur repeatedly [20, 21].

Especially in younger infants, a primary RSV infection evokes a poor immune response, and it has limited effect on subsequent reinfection [22, 23]. Once a host is exposed to RSV, the innate mucosal immune response is activated and it determines the serum antibody response that prevents the progression to LRTI through virus neutralization [24–26].

The response to RSV infection induces both serum and secretory antibody production, even in the first years of life when the antibody titers are lower [27, 28]. In murine models, RSV-specific antibodies do not seem to play an essential role in the resolution of infection [29]. Canadian databases reported that the incidence of RSV-induced LRTI is significantly lower with higher antibody titers [30]. However, when the virus infects the lungs of the host it is the cellular immune response that promotes viral clearance.

T-cell mediated immune response is also involved in the protection against RSV associated illness through the induction of viral clearance, even if the mechanisms of the immune response has not been extensively clarified [25, 26].

The role of CD4 T cells is crucial to maintain the balance between the effector and the regulatory immune response in the lungs infected by RSV. An abnormal CD4 T-cell response was observed to lead to a more severe disease through the activation of Th2-, Th9-, and Th17-related cytokines as observed in murine models or in some vaccination trials [31]. Moreover, IL-10 polymorphisms are related to susceptibility to severe LRTI [31].

RSV infection leads to a CD4 Th2-mediated immune response resulting in mucus production and airway hyperactivity that determine the symptoms of bronchiolitis and LRTI; young infants are more predisposed toward this response than adults [32].

Newborn and infants, particularly those born prematurely, are susceptible to severe LRTI because of the small size of the airways where the airway edema and sloughing of epithelial cells cause atelectasis and subsequent mismatching of ventilation and perfusion and decreased oxygenation.

CD4 Th2 cells also play a key role in the regulation of the immune response aimed to protect the lungs to an immunomediated toxic effect; this homeostasis is mediated by regulatory T cells (Tregs) and IL-10 [33–36]. Alterations in number and function of Tregs and IL-10 result in a more severe immune response after RSV infection in animal models, while the effects of these alterations in human are still not clear, but they have an influence on the clinical course of the infection [32].

Several hypotheses on the mechanisms modulating the immune response against RSV infections may be inferred from an unfortunate experience with a potential RSV vaccine that occurred in the 1960s. In the 1960s, an experimental vaccine against RSV inactivated with formalin (FI-RSV) was administered in a randomized controlled trial to a group of four-month-old infants and their siblings younger than 10 years (219 subjects received FI-RSV vaccine) and to a group of healthy infants between the ages of 6 and 24 months [36]. As a result of a subsequent natural RSV infection that occurred during the following epidemic season, 80% of the FI-RSV-vaccinated children were hospitalized with a severe LRTI [36–38]. In two cases, RSV infection after FI-RSV-immunization was fatal [39]. Postmortem histology of the lungs revealed an extensive mononuclear cell infiltrate and the presence of numerous eosinophils. In addition, a significantly greater number of eosinophils were found in the blood of vaccinated infants than in blood of control children [36]. The immune mechanisms responsible for this response to vaccine were subsequently investigated through a murine model.

The lack of protective effect of FI-RSV vaccine and its correlation to a more severe clinical course of the infection was probably due to several factors, including the inadequate development of poorly neutralizing antibodies against RSV-encoded epitopes, incomplete affinity maturation of the antibodies, and lack of an anti-RSV cytotoxic T lymphocyte response [40]. The response to FI-RSV vaccine was associated to the activation of IL-4 secreting CD4+ cells (Th2) with more significant pulmonary damages, often marked by eosinophilic infiltration, leading to severe respiratory disease in these children [41, 42].

Children with T-cell deficiencies are unable to efficiently clear RSV, confirming that T cells indeed play a role in virus neutralization and subsequently they are at a higher risk for severe and potentially lethal viral infections, including RSV LRTI [23, 43]. Moreover, both neutropenia and lymphocyte depletion are risk factors for prolonged and increased viral replication and shedding, which leads to severe and prolonged respiratory infections. The most important causes of deaths in infants with primary immunodeficiency are sepsis and pneumonia [44].

The real incidence of primary immunodeficiency is probably underestimated [45], since they usually start with recurrent infections that are very common also in healthy infants in the first period of life. Infections are often severe and caused by unusual pathogens, but also recurrent respiratory infections caused by RSV and other widespread respiratory viruses are often a presenting feature of undiagnosed T-cell immunodeficiencies at a median age of four months [46, 47].

## 3. Epidemiology of RSV Infections in Immunocompromised Infants

Clinical studies considering the incidence of RSV infections in immunocompromised infants often consider both primary and acquired immunodeficiencies, probably because RSV causes the same clinical manifestations in infants with any severe immunodeficiency including hematopoietic stem cell transplantation and oncologic disorders [8, 11, 39, 48, 49], HIV infection [50, 51].

The first study considering immunocompromised infants with RSV infections was conducted over ten consecutive winter seasons, [52] and analyzed the correlation between the severity of an episode of RSV infection and the immune status in 608 hospitalized infants younger than 5 years. Of these, 7.7% of them had an immunocompromised status due to chemotherapy, steroid therapy, or a primary immunodeficiency disorder and they had a higher mortality rate. A retrospective review [53] observed that 2.2% infants hospitalized for RSV infections were immunocompromised. Moreover, infants with immunodeficiency had a longer hospital stay (median 39 days) than other high-risk infants like those with CHD or CLD (11 days) and infants <6 weeks of age (5 days). The presence of preexisting diseases including immunodeficiencies was demonstrated to increase admissions in intensive care units (ICU) and mortality (RR 2.36, 95% CI 2.02 to 2.76) in a cohort of 406 infants hospitalized for RSV infections from 1999 to 2007 [12]. A retrospective study [43] reviewed 22 cases of respiratory infections in children with primary immunodeficiency undergoing bone marrow transplantation: RSV was detected in 3 cases of severe viral pneumonitis that were treated with ribavirin and high-titre specific immunoglobulin. In the PICNIC study (Paediatric Investigators Collaborative Network on Infections in Canada) [54], the incidence of RSV-related hospitalizations was observed to be higher in infants with immunodeficiency, as well as other high-risk infants such as infants born prematurely or with CLD or CHD. Infants with Down syndrome are at high risk of severe LRTI because they are often affected by CHD, but they may also have immunological disorders involving B cells and T cells [55–57]. A prospective national birth cohort [58] reported a hospitalization rate near 10% in these infants irrespective of coexisting CHD. This phenomenon occurred because Down syndrome children have altered immune response, abnormal thymic development and function, and decreased number of B cells and T cells, especially in the first 2 years of life. In addition,* in vivo* proliferative tests showed an increased susceptibility to infectious pathogens. These data were confirmed by a recent cohort study [7] that enrolled 117 immunocompromised pediatric in-patients with an acute community-acquired RSV infection from 2006 to 2011: 28.2% had Down syndrome. The ICU admission rate was >20% and the mortality rate was 5%.

## 4. RSV Prophylaxis in Immunocompromised Infants

Environmental prevention is extremely important to reduce both nosocomial and community-acquired respiratory infections, in particular in high-risk patients as immunocompromised infants.

The diffusion of the virus can be controlled by protective measures such as hand washing, isolation of positive hospitalized cases (cohorting) and with the use of gloves, masks, and protective clothing by dedicated staff. Moreover, in epidemic seasons immunocompromised infants and children should be hospitalized in special designated areas where other patients, caregivers, and visitors should be checked for respiratory infections and where the admission of visitors is limited [59].

Pharmacological prophylaxis in immunocompromised infants is based on the use of palivizumab. Palivizumab is a humanized IgG1 monoclonal antibody obtained through recombining DNA technique specific for an epitope of antigen A of F (fusion) protein of RSV. The mAbs have the same properties of a human IgG1, with a long half-life (28 days). It is free from the risk of transmission of blood-borne pathogens, which is particularly important in immunocompromised infants. Palivizumab was approved by the Food and Drug Administration in the United States in 1998; it is currently the only approved monoclonal antibody used for RSV prophylaxis and it was observed to determine a 55% decrease in RSV-related hospitalizations [60]. It is composed of two sequences, a human one (95%) and a murine one (5%). Palivizumab neutralizes and inhibits the fusion of the F protein of the virus to infected epithelial respiratory cells and interferes with viral replication. It is currently used in epidemic periods with monthly intramuscular administration to prevent RSV hospitalizations in high risk subjects. The use of this drug is regulated by guidelines and recommendations of several pediatric societies to optimize its cost-effectiveness [61–66] (AAP, Canadian Pediatric Society, Italian Neonatology Society, Spanish Neonatology Society, Japan Pediatric Society, and others). The recommendations are well defined in presence of prematurity, CLD, and hemodynamically significant CHD. However, the use of this drug in infants with immunological disorders is instead still controversial and rarely recommended in current guidelines and its cost-effectiveness is still a matter of debate. Until recently, the British Columbia Guidelines [65] has stated that infants eligible for palivizumab administration include infants with severe immunodeficiency. A recent Japanese study [67] reported that 1,115 infants younger than 4 years were hospitalized for RSV infections in a 2-year consecutive seasons: 756 of them had underlying disorders that were not included in the criteria for palivizumab prophylaxis in Japan. These disorders included severe immunodeficiency and Down syndrome.

The severity of RSV infections in infants with primary immunodeficiency and the lack of recommendations for prophylaxis in current guidelines have led several authors to consider the “off-label” extension of the use of palivizumab to these uncommon and severe diseases. A Delphi study was performed by Gaboli et al. [9] to obtain a consensus in a group of expert pediatric pulmonologists regarding the off-label use of palivizumab in 43 diseases: the consensus was obtained for 24 out of the 43, including primary immunodeficiencies. In Italy, even if prophylaxis in these cases was not included in the Italian Neonatology Society [62] recommendations, some authors [68] administered the prophylaxis with palivizumab to 2 infants with primary immunodeficiencies (one case with Di George syndrome and one case with Wiskott-Aldrich syndrome) and 2 infants with acquired immunodeficiencies (2 cases with HIV infection) with good compliance and efficacy.

## 5. Conclusions

Mortality and morbidity due to RSV LRTI infections are higher in infants with primary immunological disorders than in healthy infants. The risk is similar to other rare and severe disorders such as malformations of the airways, cystic fibrosis, and neuromuscular diseases, which are also not included in current recommendations for prophylaxis. The experience of clinicians treating these rare and severe disorders is essential to assess that prophylaxis should be extended to all infants at increased risk for severe and potentially lethal RSV infections.

The use of palivizumab in addition to environmental prophylaxis in selected immunocompromised patients was proved to be effective and free from adverse events. Current guidelines for this prophylaxis do not contain recommendations for these high risk infants, although some authors endorse the extension for treatment to immunodeficient subjects. The inclusion of such very high risk patients in guidelines will prevent unnecessary hospitalizations and deaths.

At present, randomized clinical trials (RCT) on the efficacy of palivizumab prophylaxis in immunocompromised infants have not been conducted, probably because of the low incidence of these disorders and the ethical controversies surrounding them.

RCT conducted in patients with immunological disorders could confirm the efficacy and the cost-effectiveness of prophylaxis with palivizumab in these infants, allowing the inclusion of infants and children with primary immunodeficiency in international recommendations and guidelines.
